# An insight into anti-adipogenic properties of an *Oroxylum indicum* (L.) Kurz extract

**DOI:** 10.1186/s12906-020-03111-2

**Published:** 2020-10-20

**Authors:** Tanaporn Hengpratom, Gordon M. Lowe, Griangsak Eumkeb

**Affiliations:** 1 School of Preclinic, Institute of Science, Suranaree University of Technology, Nakhon Ratchasima, 30000 Thailand; 2 School of Pharmacy and Biomolecular Sciences, Liverpool John Moores University, Liverpool, L3 3AF UK

**Keywords:** *Oroxylum indicum* extract, Adipogenesis, 3 T3-L1, Glucose transporter 4, Mitochondrial membrane potential

## Abstract

**Background:**

*Oroxylum indicum* fruit extract (OIE) has been reported to inhibit the development of adipocytes. However, the exact mechanism of its metabolic activity is not clearly defined. This study attempted to investigate whether OIE was involved in disrupting the cell cycle, glucose metabolism, and mitochondrial function in 3 T3-L1 cells.

**Methods:**

The effect of the OIE on cell cycle progression was measured by flow cytometry along with observing the expression of the cycle regulator by immunoblotting. The effect of the OIE on glucose metabolism was investigated. The amount of glucose uptake (2-NBDG) influenced by insulin was determined as well as the protein tyrosine phosphorylation (PY20), and glucose transporter4 (GLUT4) expression was determined by immunoblotting assay. Mitochondria are also essential to metabolic processes. This study investigated mitochondrial activity using fluorescent lipophilic carbocyanine dye (JC-1) and mitochondria mass by MitoTracker Green (MTG) staining fluorescence dyes. Finally, cellular ATP concentration was measured using an ATP chemiluminescence assay.

**Results:**

Treatment with OIE plus adipogenic stimulators for 24 h arrested cell cycle progression in the G2/M phase. Moreover, 200 μg/mL of OIE significantly diminished the expression of the insulin receptor (IR) and GLUT4 protein compared to the untreated-adipocytes (*P* < 0.05). The mitochondrial membrane potential (MMP) was significantly reduced (24 h) and increased (day 12) by OIE compared to untreated-adipocytes (*P* < 0.05). However, OIE maintained MMP and ATP at a similar level compared to the pre-adipocytes (day 12). Transmission electron microscope (TEM) results demonstrated that OIE could protect mitochondria deformation compared to the untreated-adipocytes.

**Conclusion:**

These results suggest that the inhibitory effect of the OIE on adipogenesis may potentially inhibit the cell cycle and phosphorylation of IR, leading to a decrease in glucose uptake to the cells. The OIE also slows down the mitochondrial activity of the early phase of cell differentiation, which can also inhibit the development of fat cells.

**Supplementary information:**

The online version contains supplementary material available at 10.1186/s12906-020-03111-2.

## Background

3 T3-L1 pre-adipocytes differentiation model has been extensively used for the study of adipogenesis [1]. The progression of 3 T3-L1 pre-adipocytes to adipocytes is initiated by various stimulators, which include 3-isobutyl-1-methylxanthine (IBMX), dexamethasone (DEX), and insulin. Once the cells receive these stimulators, they proliferate to confluence. Post-confluent pre-adipocytes become growth-arrested at the G1/S phase of the cell cycle [2]. The re-entry of growth-arrested pre-adipocytes into the cell cycle and the completion of several rounds of clonal expansion are the initial steps in the process of adipogenesis [3].

Adipocytes differentiation is marked by a change in the expression of many receptors on the plasma membrane. One such change is the increased expression of receptor glucose transporters, particularly GLUT4 [4]. Binding of the insulin to its receptor initiates a signaling cascade results in the translocation of GLUT4 to the plasma membrane, which leads to the facilitated diffusion of glucose into the cell [5]. Additionally, most of the adenosine triphosphate (ATP) synthesized during glucose metabolism is produced in the mitochondria through oxidative phosphorylation [6]. The previous study found that 20 to 30 fold of mitochondrial protein was increased during the differentiation of 3 T3-L1 cells, together with increased expression of lipogenic enzymes [7]. As mentioned, glucose synergizes with insulin and mitochondria function, and all three are an important factor for the lipogenic pathway.

The OIE was previously reported as having anti-adipogenic properties. Studies revealed that when pre-adipocytes were subjected to the adipogenic stimulators along with 200 μg/mL of OIE during the early phases of the adipocyte differentiation process, it significantly inhibited the accumulation of lipids and prevented the transformation to adipocytes [8]. These results led us to believe that OIE may influence the preliminary stages of adipocyte development. OIE can inhibit the expression of Peroxisome proliferator-activated receptor γ2 (PPARγ2), leading to decreased expression of adipokine and lipogenic enzymes [9]. This study intended to investigate the role of OIE on the cell cycle, glucose metabolism, and mitochondrial function in the 3 T3-L1 cell during adipogenesis.

## Methods

### Plant collections, authentication, and liquid chromatography-mass spectrometry (LC-MS) characterization

The fruits of *Oroxylum indicum* (L.) Kurz, family Bignoniaceae (*O. indicum*) were purchased from the Wang Nam Khiao District, Nakhon Ratchasima province, Thailand, from July to September 2015. Herbarium voucher specimens from all parts of this plant were prepared and authenticated by Dr. Santi Wattatana, a lecturer and a plant biologist at the Institute of Science, Suranaree University of Technology, Thailand. The voucher specimens (SOI0808U) were deposited at Suranaree University of Technology. Fresh fruits of *O. indicum* with seeds were cut into small pieces and dried in the oven at 40 °C. Then, air-dried fruits were ground into a powder with an electric grinder. 500 g of dry powder was extracted with 95% ethanol by a soxhlation for 8 h. The extract was then filtered through Whatman No. 1 filter paper. Next, the ethanolic filtrate was concentrated using a vacuum rotary evaporator (50 °C) and dried by lyophilization to give crude dried extracts of *O. indicum*. Crude dried extracts were stored at − 20 °C until use. In cell culture experiments, the OIE was dissolved in 100% dimethyl sulfoxide (DMSO) and then diluted to 0.1% (v/v) DMSO in cell culture medium when preparing the designated concentrations (50 to 200 μg/mL).

For LC-MS analysis, the constituents of the whole fruit OIE had been investigated following the method of Hengpratom et al. with some modifications [9]. Briefly, the OIE was dissolved in the methanol at concentrations 20 and 10 mg/mL. The experiment was performed on the Dionex Ultimate 3000 UHPLC system (Dionex, USA) coupled with electrospray ionization (ESI) tandem mass spectrometer (micro-TOF-QII) (Bruker, Germany). The optimum separation was achieved with a Zorbax SBC18 column (250 mm × 4.6 mm × 3.5 μm) (Agilent Technologies, USA) with a flow rate of 0.8 mL/min of the mobile phase, which comprised 0.1% formic acid in deionized water (solvent A) and acetonitrile acid (solvent B). The gradient program was starting from 30% B to 80% B in 30 min and stayed at 80% B until 38 min, then the gradient program was adjusted to 30% B in 2 min and stayed until the run ending at 45 min. The injection volume of all samples was 5 μL. The results were obtained in a mass scanning mode in the range of 50 m/z to 1500 m/z at negative ion polarity. Nebulizer gas was 2 Bar, dry gas, and temperature were set to 8 L/min and 180 °C; the capillary voltage was 4.5 kV. The calibration curves of the reference standards were obtained from the concentration ranges between 0.5 μg/mL to 250 μg/mL, and the concentration of targeted compounds was calculated using the equation for linear regression obtained from the calibration curves.

### Adipocytes differentiation and treatment

The 3 T3-L1 pre-adipocytes cell line was purchased from American Type Culture Collection (ATCC, Manassas, VA, USA). The differentiation protocol was derived from a previous study [10]. At 2 days following confluence (day 0), the cells were stimulated to differentiate with Dulbecco’s Modified Eagle Medium (DMEM) containing 10% fetal bovine serum (FBS) (Hyclone, Logan, UT, USA), 1.0 μM of dexamethasone (G Bioscience, St. Louis, MO, USA), 0.5 mM of IBMX and 1.0 μg/mL of insulin for 2 days. From day 4 onwards, the differentiation media were replaced by 10% FBS/DMEM media containing 1.0 μg/mL of insulin. These media were changed every 2 days until the cells were harvested. All media contained 100 μg/mL of streptomycin and 100 U/mL of penicillin (GIBCO). Cells were maintained at 37 °C in a 95% humidified with 5% of CO_2_ atmosphere.

For the treatment of 3 T3-L1 cells, the cells were seeded in a 6-well plate at the density of 1.5 × 10^5^ cells/well. The cells were allowed to adhere to the plate for 48 h and were then divided into 6 groups; 1) non-differentiated cells (pre-adipocytes, ND); 2) differentiated cells treated with 0.1% DMSO (untreated-adipocytes, D); 3–6) differentiated cells treated with 50, 100, 150, and 200 μg/mL OIE (OIE-treated adipocytes, D + OIE), respectively. The different concentrations of OIE were diluted to get 0.1% (v/v) DMSO in cell culture media and treated throughout the differentiation period until the cells were harvested. Twenty-four hours after the cells were induced to differentiate. The cells were collected for cell cycle analysis and protein quantification. For additional experiments, the cells were collected on day 12 for protein quantification**,** immunocytochemistry, flow cytometry, and TEM analysis.

### Cell cycle analysis

Post confluent 3 T3-L1 pre-adipocytes were stimulated by the differentiated media in the absence and presence of OIE (50 to 200 μg/mL) for 24 h. The cells were collected by trypsinization and washed twice with phosphate-buffered saline (PBS). The cells were then fixed in 80% of ethanol and stored at − 20 °C for 1 h. The fixed cells were washed twice with ice-cold PBS. The cells were treated with 50 μL of RNaseA (0.1 unit/mL) and 150 μL of Propidium iodide (PI) (50 μg/mL) at 37 °C for 1 h. The fluorescence intensity of the PI stained cells was measured using the BD Accuri C6 flow cytometer. The data from 25,000 cells per sample were analyzed using FlowJo software and the Dean-Jett-Fox model.

### Protein extraction and immunoblot analysis

The expression of cyclin-dependent kinase (Cdk2), PY20, and GLUT4 proteins in control and OIE-treated cells was determined by Western immunoblotting. The lysates (50 μg, each) were separated on 12% Mini-PROTEAN TGX stain-free pre-cast gels. After electrophoresis, the proteins were transferred to polyvinylidene difluoride (PVDF) using the Trans-Blot Turbo system (Bio-Rad, Watford, UK). The membranes were then blocked with 5% skimmed milk for 1 h at room temperature, followed by a washing step. The membranes were subsequently incubated with mouse monoclonal antibody (anti-Cdk2, anti-PY20, and anti-GLUT4) (1:1000 in PBS), overnight at 4 °C. After extensive washing, the membranes were incubated with mouse IgGκ light chain binding protein (m-IgGκ BP) conjugated to horseradish peroxidase (HRP) (1:3000 in PBS) for 1 h at room temperature, followed by washing with PBS with 0.1% Tween 20 (PBST). The antigen-antibody complex was developed using enhanced chemiluminescence (ECL) substrate solution for 5 min (Bio-Rad, Watford, UK). The intensities of the adiponectin protein bands were quantified using ImageJ software. The data were normalized using *β*-actin as an internal control.

### Immunocytochemistry

On day 12, 3 T3-L1 pre-adipocytes and adipocytes were collected, washed with cold PBS, fixed for 15 min in cold acetone, and washed with PBST. After blocking with 4% of bovine serum albumin (BSA) for 1 h at room temperature, the cells were washed and incubated with anti-GLUT4 antibody (1:200 in PBS) overnight at 4 °C. After extensive washing, mouse IgGk conjugated with fluorescein isothiocyanate (FITC) (1:500) was applied to the samples and incubated for 90 min at room temperature. After washing thoroughly with PBS, the cells were double-stained with 4′,6-Diamidino-2-Phenylindole (DAPI) (nucleus stain) (1:1000) for 5 min in the dark and washed twice with PBS. Finally, the cells were visualized using fluorescence microscopy (Leica DMI6000B).

### Flow cytometry

Flow cytometry was used to detect glucose concentration and mitochondria activity in 3 T3-L1 cells. At 24 h after inducing cells with the differentiation media and on day 12, the cells were collected and washed twice with PBS. Then, cells were incubated with fluorescence dye for 60 min, including 0.1 mM of 2-NBD-Glucose (2-NBDG) for glucose uptake and 10 μM of JC-1 for MMP. Next, cells were washed twice with PBS. Labeled cells were collected (keep in the dark) and analyzed using a flow cytometer with excitation 488 nm. All data were recorded and analyzed using BD accuri C6 software. The data were presented as the median fluorescent signals for 10,000 cells.

### Confocal microscopy

The MitoTracker green fluorescent dye was used to label mitochondria within 3 T3-L1 cells. On day 12, the culture media were removed, and the cells were incubated with 200 nM of MitoTracker green fluorescent dye for 45 min at 37 °C. Then, the cells were carefully washed with culture media and trypsinized. Labeled cells were separated and deposited onto a Shandon cytoslide glass slide by using Shandon cytospin 4 cytocentrifuge, following manufactured protocol (Thermo Fisher Scientific). Briefly, 200 μL of each sample was loaded in the Shandon cytofunnel chamber and spun at 1000 rpm for 5 min to allow for complete fluid absorption. The cells were allowed to dry at room temperature, and the mounting medium was applied to the surface of the slide along with a cover slide. The images were obtained using a Zeiss 510 Meta laser scanning microscope mounted on an Axiovert 200 M BP computer-controlled inverted microscope. The Argon ion laser was used with an excitation wavelength of 488 nm, and the images were captured at × 100 and × 200 magnification.

### ATP measurement

ATP level was measured as previously described with some modifications [11]. On day 12, the media were removed, and the cells were washed twice with 1 M of cold PBS. The level of ATP released was then determined using the ATP bioluminescent somatic cell assays kit (Sigma-Aldrich, Dorset, UK). Fifty microliter of ATP releasing reagent stock with 450 μL of ultrapure water was added directly to the cells and incubated for 5 min. Then, 20 μL of mixed samples were taken to the 96 well plates, followed by adding 80 μL of ultrapure water. Finally, 100 μL of the luciferase assay mix was added to the sample. The amount of light emission was then measured using a microplate luminometer. ATP concentration (0 to 200 nmol/L) was determined from the standard curve. The protein concentrations in each well were used to normalize the ATP content (nM/mg of protein).

### Transmission electron microscopy

On day 12, the cells were dissociated with 0.25% trypsin at 37 °C for 3–5 min, and the digestion was stopped with a culture medium containing FBS. The cells were transferred into a 10 mL tube, centrifuged at 1200 rpm for 8 min. They were washed with PBS (4 °C) and then centrifuged at 1200 rpm for a further 8 min. After discarding the supernatant, 2.5% glutaraldehyde in 0.1 M phosphate buffer was added to the cells and left to fix at 4 °C overnight. The cells were rinsed three times with 0.1 M phosphate buffer (pH 7.2) for 15 min each, followed by post-fixation with 1% Osmium tetroxide (OsO_4_) prepared in dH_2_O. Fixed samples were dehydrated, embedded in epoxy resin (Electron Microscopy Sciences), and polymerized at 60 °C for 24 h. The blocks were ultra-thin-sectioned at 60 nm with a diamond knife using an RMC ultra-microtome. The sections were placed on copper grids and stained with 2% uranyl acetate at room temperature for 15 min and then rinsed with distilled water, followed by secondary staining with lead stain solution (SPI-CHEM) at room temperature for 15 min. The grids were observed under a TEM (FEI Model TECNAI G2 20S-TWIN) at an acceleration voltage of 120 kV.

### Statistical analysis

All the data were expressed as the mean ± standard deviation of the mean (Mean ± SD). The difference values between cell population in the cell cycle, Cdk2 expression, PY20, and GLUT4 proteins, glucose metabolism uptake, mitochondrial activity, MMP, and ATP production compared between groups were analyzed using one-way analysis of variance (ANOVA) with a Tukey’s HSD post-hoc test (SPSS v 23). Values were considered statistically significant when *P* < 0.05. The experiments were performed in three independent experiments (each run triplicate). However, the experiments of cells staining (Figs. 5, 7, and 10) and TEM (Fig. 11) were performed in two independent experiments (each run triplicate).

## Results

### Liquid chromatography-mass spectrometry analysis

**Fig. 1:**
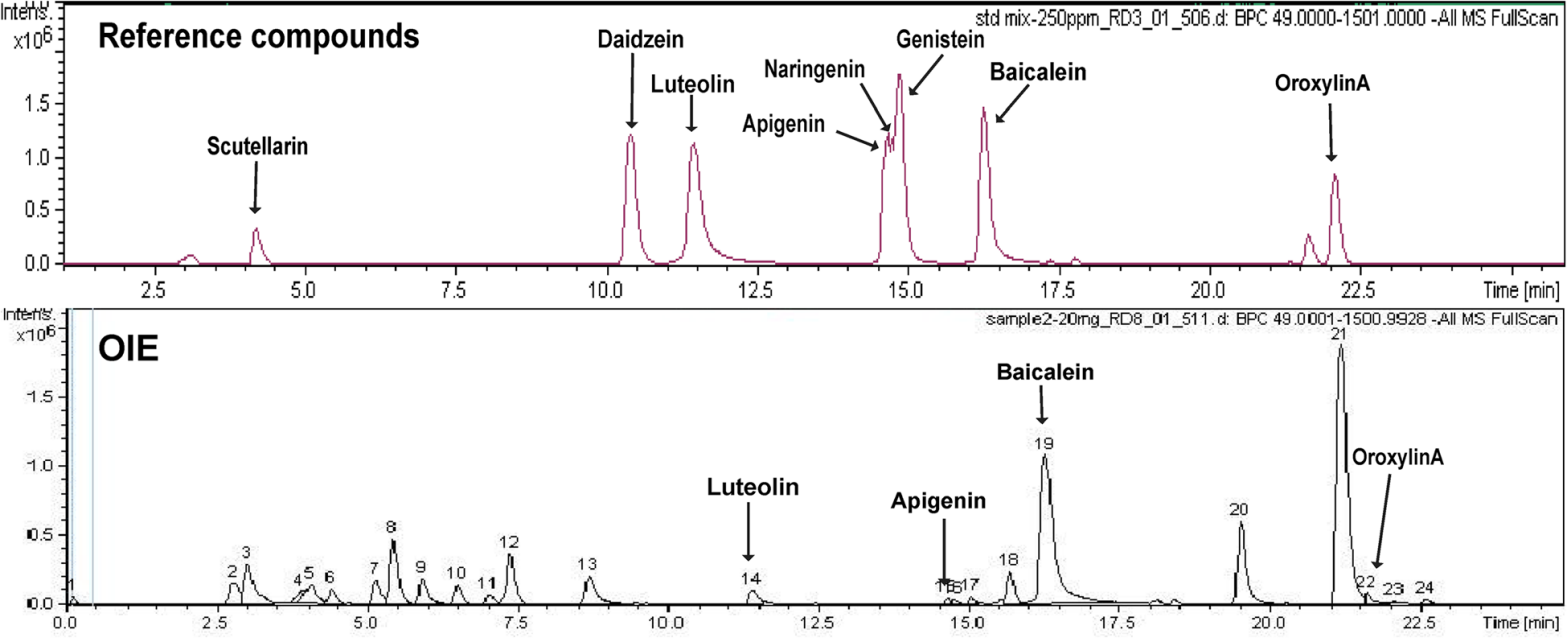
LC-MS chromatograms of OIE at different retention time correspondences to the mass spectra of luteolin, apigenin, baicalein, and oroxylin A, respectively

This study examined the presence of key flavones and associated compounds in OIE. The mass spectral data of the reference compounds and sample can be seen in Fig. 1 and the content in Table 1. The major components of OIE had composed of 1) luteolin 2) apigenin 3) baicalein, and 4) oroxylin A (Fig. 1). The profiles of these compounds were compared to authentic standards. The most dominant flavonoid in OIE was baicalein; indeed, 20 mg/mL of OIE contains 509.96 μg/mL of baicalein (Table 1). In addition, low amount of oroxylin A (5.33 μg/mL), luteolin (4.20 μg/mL), and apigenin (1.55 μg/mL) were also detected in OIE. Moreover, there are two major unknown compounds, including unknown1 (peak 20) and unknown2 (peak 21) (Fig. 1, Table 1). The spectral result of peak 21 showed at m/z 254, which is consistent with the standard chrysin peak [13]. In the same way, the study of Rojsanga reported that *O. indicum* seed extract contained 3 major compounds, including baicalein, baicalin, and chrysin [14]. Thus, a peak 21 could be the chrysin. However, further investigation is needed.

**Table 1:** Quantitative contents of the main compounds in *O. indicum* fruit extract determined by LC-MS

Reference standard	Retention time [12]	Quantitation mass (m/z)	Concentration in 20 mg/mL of OIE (μg/mL)
Scutellarin	4.2	285	ND
Daidzein	10.4	253	ND
Luteolin	11.4	285	4.20
Apigenin	14.6	269	1.55
Naringenin	14.7	271	ND
Genistein	14.8	269	ND
Baicalein	16.2	269	509.96
Unknown1	19.5	538	–
Unknown2	21.1	254	–
Oroxylin A	22.0	283	5.33

*ND* not detected

### Effect of OIE on inhibition of cell cycle progression

**Fig. 2:**
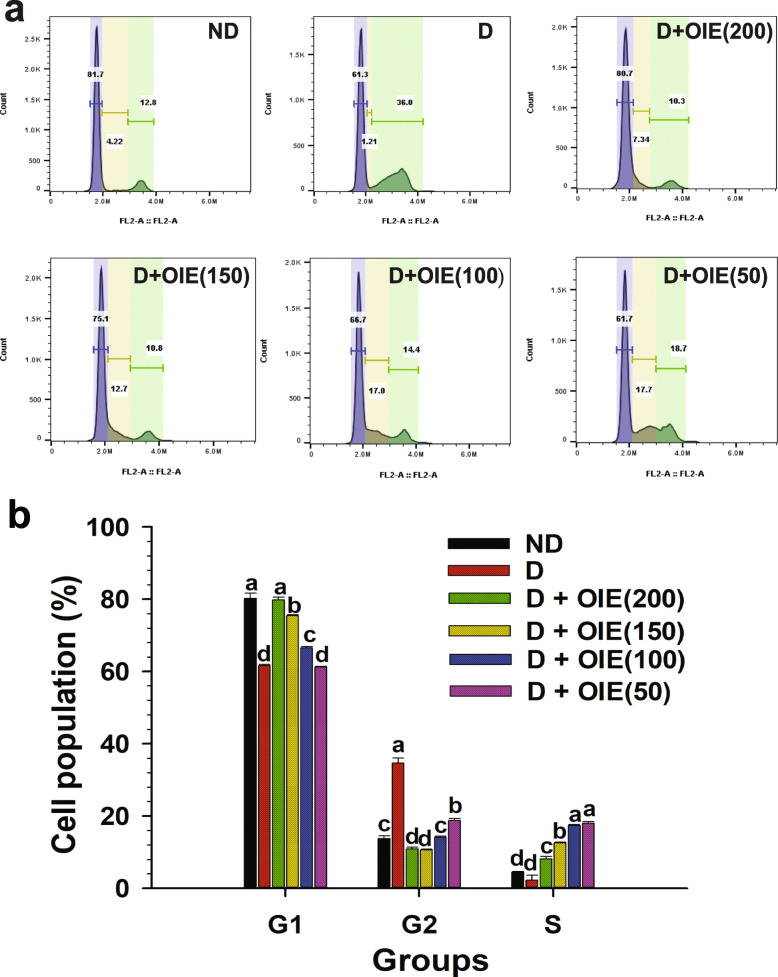
The effect of the OIE on the cell cycle. 3 T3-L1 cells were treated by differentiation media in the presence of OIE at the concentration of 50 to 200 μg/mL for 24 h. The cells were stained with PI and analyzed by flow cytometry for the determination of the cell population. **a** Flow cytometry data of the cell population in the G0/G1, S, and G2/M phases was determined by Flow Jo software. **b** The graph represents the percentage of the cell population. Means ± SD value (*n* = 9) of three independent experiments is displayed. ND: non-differentiated cells (pre-adipocytes); D: differentiated cells with 0.1% DMSO (untreated-adipocytes); D + OIE(200): differentiated cells with OIE at 200 μg/mL (OIE-treated adipocytes). Differences among groups were determined by one-way ANOVA followed by Tukey’s Post-hoc test, and the different superscript alphabets are significantly different from each other (*P* < 0.05)

**Fig. 3:**
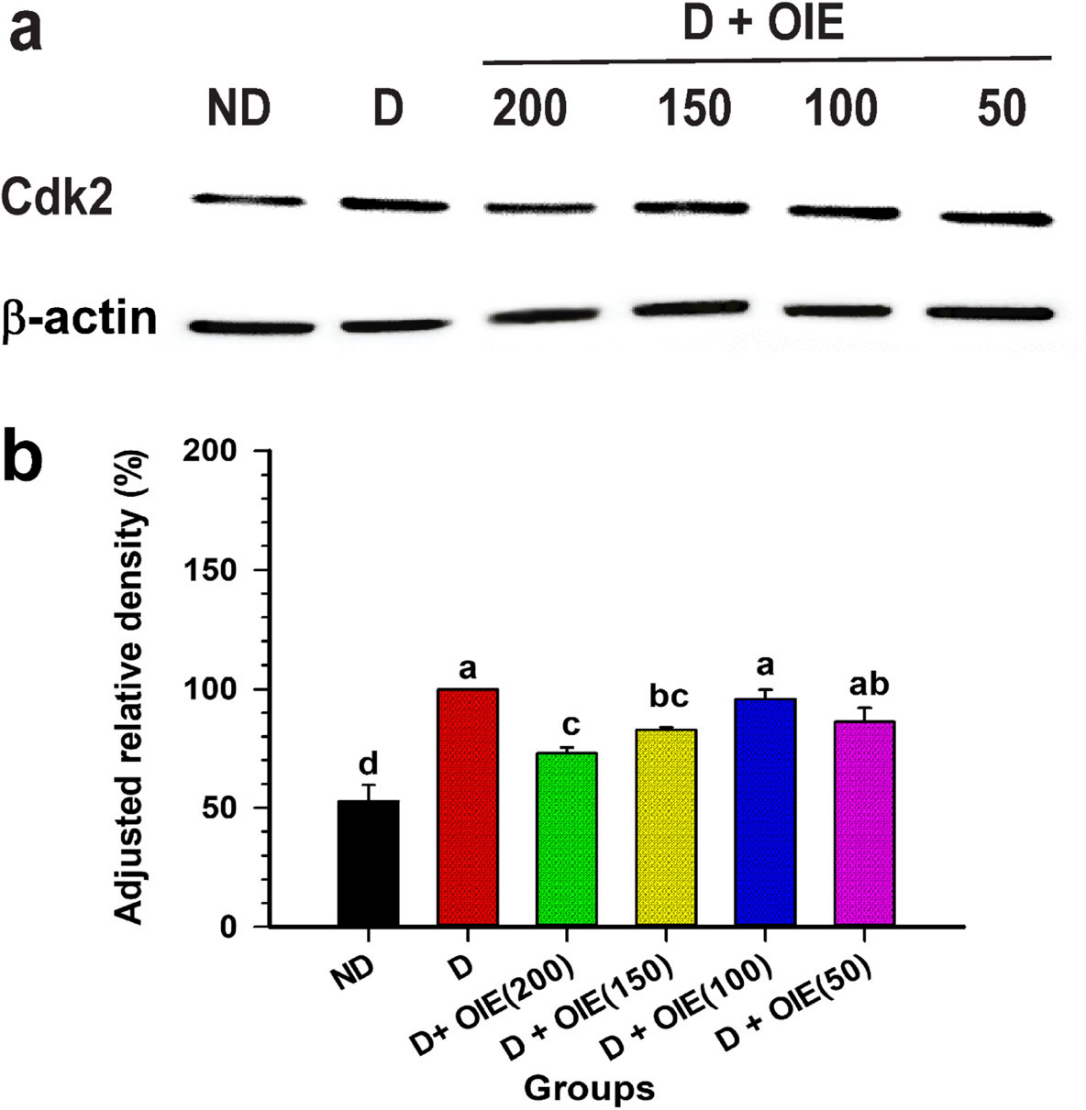
The effect of the OIE on the expression of Cdk2 in 3 T3-L1 cells. **a** Western blot analysis of the expression of Cdk2 in cells treated with OIE at a concentration range from 50 μg/mL to 200 μg/mL at 24 h. The total lysates of 3 T3-L1 cells with the treatment mentioned above were resolved on Mini-PROTEAN TGX gels followed by transfer to PVDF membrane and probed with the anti-Cdk2 antibody (primary antibody) and secondary antibody. The protein was visualized using chemidoc with the ECL detection kit. **b** The densitometry analysis of Western blot bands was normalized against β-actin and expressed as a mean of ± SD of three independent experiments (*n* = 9). ND: non-differentiated cells (pre-adipocytes); D: differentiated cells with 0.1% DMSO (untreated-adipocytes); D + OIE(200): differentiated cells with OIE at 200 μg/mL (OIE-treated adipocytes). Differences among groups were determined by one-way ANOVA followed by Tukey’s Post-hoc test, and the different superscript alphabets are significantly different from each other (*P* < 0.05). The full-length of the blots are presented in Supplementary Figure 1

The effect of the OIE on the cell cycle of 3 T3-L1 cells is shown in Fig. 2a and b. When 3 T3-L1 pre-adipocytes were induced to differentiate with adipogenic stimulators, the cells enter into S and G2/M phase within 24 h following induction (Fig. 2a). Further analysis revealed that differentiated cells transformed from G1 and S phase to the G2 phase (36% cells in G2 phase). In contrast, treatment with OIE at concentrations ranging from 50 to 200 μg/mL significantly arrested cells in the G0/G1 phase (10.3, 10.8, 14.4, and 18.7% cells in G2 phase, respectively) compared to controls (*P* < 0.05) (Fig. 2a and b). The impact of the OIE on the cell cycle was further explored, and the expression of Cdk2 in differentiating cells was affected at the doses of 150 and 200 μg/mL (Fig. 3a and b). These results suggest that OIE may inhibit cell cycle entry into the G2/M phase of 3 T3-L1 cells. OIE has the potential to inhibit the initiation of mitotic clonal expansion (MCE), which is a significant step for cell differentiation. Thus, this mechanism may play an important role in the suppression of adipogenesis.

### Effect of OIE on glucose metabolism

**Fig. 4:**
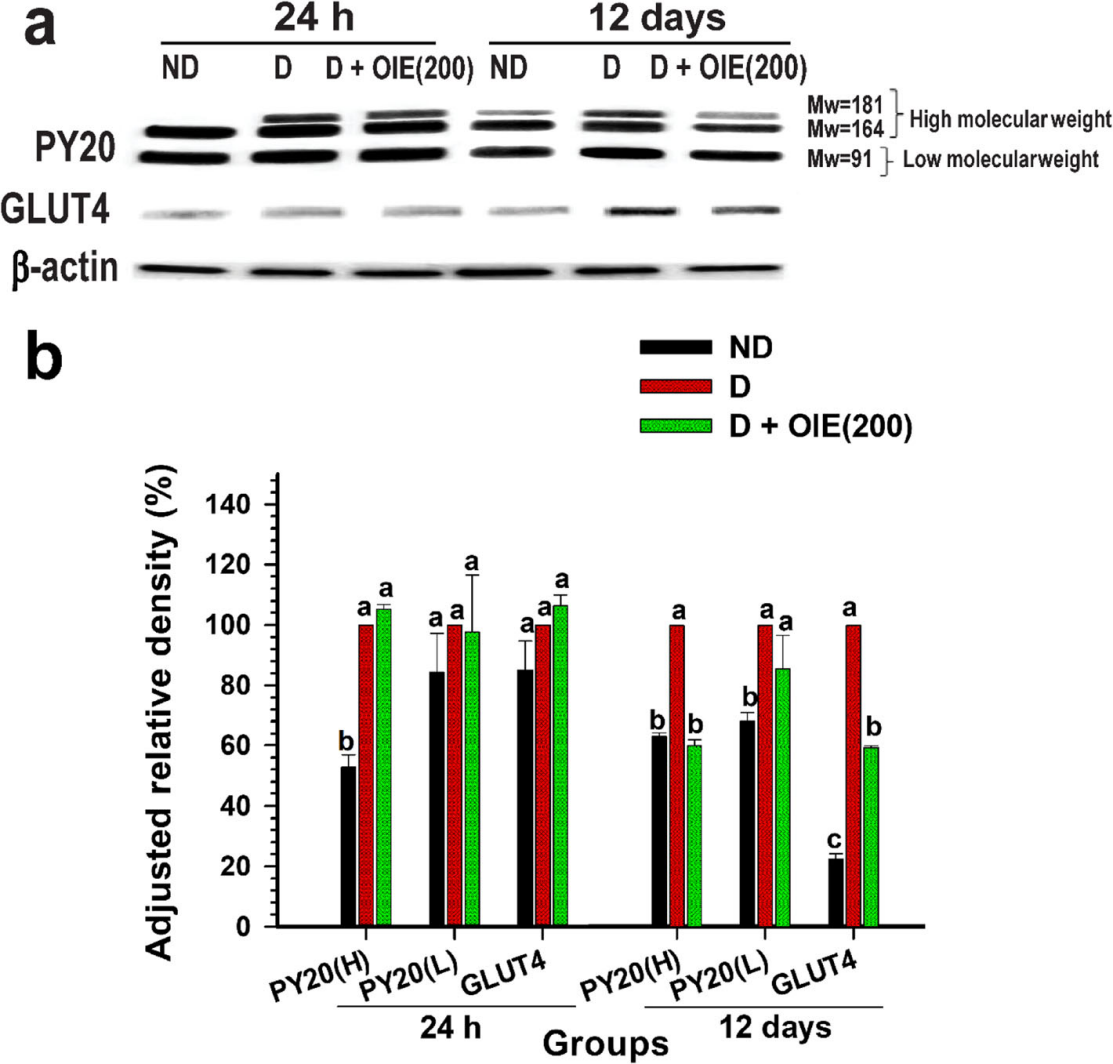
The effect of OIE on glucose metabolism in 3 T3-L1 cells. **a** Western blot analysis displays the expression of PY20 and GLUT4 in cells treated with 200 μg/mL OIE at 24 h and 12 days. The total lysates of 3 T3-L1 cells were resolved on Mini-PROTEAN TGX gels followed by transfer to a PVDF membrane, probed with the anti-phosphotyrosine antibody, anti-glucose transporter 4 (primary antibody), and a secondary antibody. The protein was visualized using chemidoc with the ECL detection kit. **b** The densitometry analysis of Western blot bands was normalized against β-actin. Means ± SD value (*n* = 9) of three independent experiments is presented. PY20(H) = protein tyrosine phosphorylation with 181 or 164 kDa; PY20(L) = protein tyrosine phosphorylation with 91 kDa; ND: non-differentiated cells (pre-adipocytes); D: differentiated cells with 0.1% DMSO (untreated-adipocytes); D + OIE(200): differentiated cells with OIE at 200 μg/mL (OIE-treated adipocytes). Differences among groups were determined by one-way ANOVA followed by Tukey’s Post-hoc test, and the different superscript alphabets are significantly different from each other (*P* < 0.05). The full-length of the blots are presented in Supplementary Figure 2

**Fig. 5:**
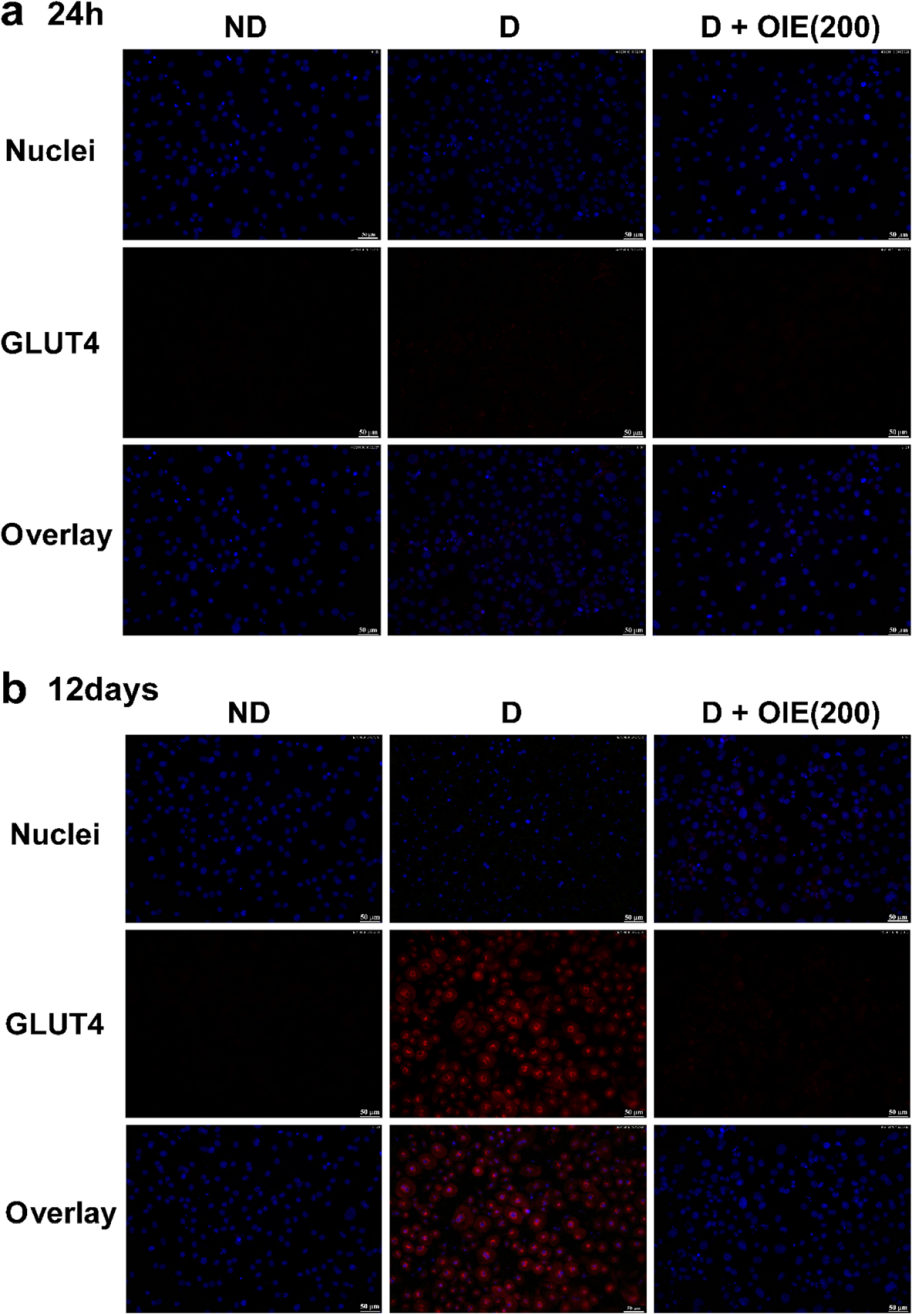
The effect of OIE on GLUT4 expression in the 3 T3-L1 cell. 3 T3-L1 pre-adipocytes, adipocytes, and OIE treated-cells (200 μg/mL) were collected at 24 h and day 12. The cells were incubated with anti-GLUT4 antibody (primary antibody) and mouse IgGk (secondary antibody). Then, the cells were double-stained with DAPI (nucleus stains). **a** The pictures were taken at 24 h, representing cellular and nuclear morphology of the cells, Nuclei (blue), GLUT4 (red), and co-immunostaining (overlay all channels). **b** The pictures were taken on day 12, representing cellular and nuclear morphology of the cells, Nuclei (blue), GLUT4 (red), and co-immunostaining (overlay all channels). Scale bar; 50 μm. Two independent experiments (each run triplicate) were performed. ND: non-differentiated cells (pre-adipocytes); D: differentiated cells with 0.1% DMSO (untreated-adipocytes); D + OIE(200): differentiated cells with OIE at 200 μg/mL (OIE-treated adipocytes)

**Fig. 6:**
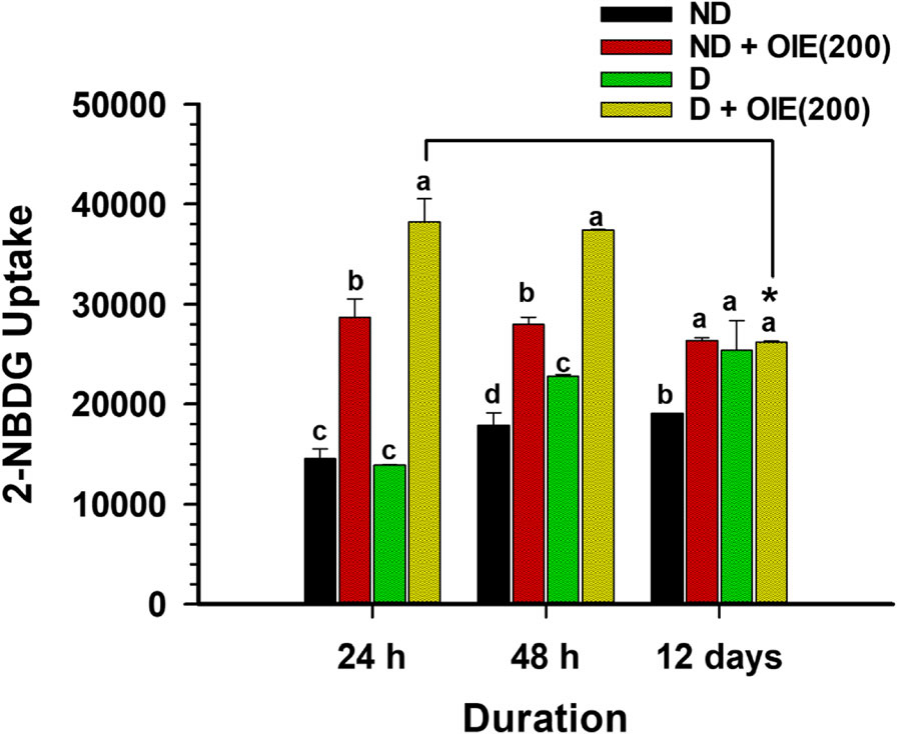
The effect of OIE on glucose metabolism uptake in 3 T3-L1 at 24 h; 48 h; and 12 days. The cells were incubated with 2-NBDG fluorescence dye for 60 min at different periods. Labeled cells were collected and analyzed using a flow cytometer with excitation 488 nm. Means ± SD value (*n* = 9) of three independent experiments is shown. ND: non-differentiated cells (pre-adipocytes); D: differentiated cells with 0.1% DMSO (untreated-adipocytes); D + OIE(200): differentiated cells with OIE at 200 μg/mL (OIE-treated adipocytes). Differences among groups were determined by one-way ANOVA followed by Tukey’s Post-hoc test; the different superscript alphabets are significantly different from each other (*P* < 0.05). The differences of glucose uptake between D + OIE during 24 h versus 12 days were analyzed by the Paired *t*-test, * *P* < 0.05

The effect of the OIE on glucose metabolism was determined by evaluating the expression of GLUT4, the impact on the 2-NBDG uptake, and the PY20 in differentiating 3 T3-L1 cells. The addition of 1.0 μg/mL of insulin in the media-induced the tyrosine phosphorylation of three major proteins with apparent molecular masses of 181 (PY20(H)), 164 (PY20(H)), and 91 kDa (PY20(L)), respectively at both 24 h and 12 days following differentiation (Fig. 4a and b). These proteins were consistent with insulin activity [15, 16]. However, phosphorylation proteins PY20(L) and GLUT4 at 24 h showed no significant difference among the pre-adipocytes, untreated-adipocytes, and the 200 μg/mL of OIE (*P* > 0.05). After 12 days, there were significantly reduced on the GLUT4 and PY20(H) of OIE-treated compared to the untreated-adipocytes. At the same time, the PY20(H) level of OIE-treated was not different from the pre-adipocytes (*P* > 0.05), while the level of PY20(L) of OIE-treated was significantly higher than pre-adipocytes (*P* < 0.05). It was thought that reduced insulin activity might influence the expression of GLUT4. By day 12 in untreated adipocytes, there was a significant expression of GLUT4 on the plasma membrane (Fig. 5a and b). Treatment with OIE resulted in a 40% reduction of the GLUT 4 expression compared to untreated adipocytes (Fig. 4a and b). In a parallel experiment, both pre-adipocytes- and adipocytes-treated with OIE at 200 μg/mL were approximately two folds significantly increased uptake of 2-NBDG compared to both untreated adipocytes at 24 and 48 h (*P* < 0.05, Fig. 6). However, on day 12, OIE-treated adipocytes were significantly reduced compared to the same groups at 24 h and 48 h (*P* < 0.05). One explanation for this is that OIE may diminish the activity of tyrosine kinases leading to a reduction of GLUT4 expression in the plasma. Moreover, the OIE may use the other facilitative glucose transporter, GLUT1 (insulin-independent), which is mostly expressed in pre-adipocytes [17].

### Effect of OIE on mitochondrial activity

**Fig. 7:**
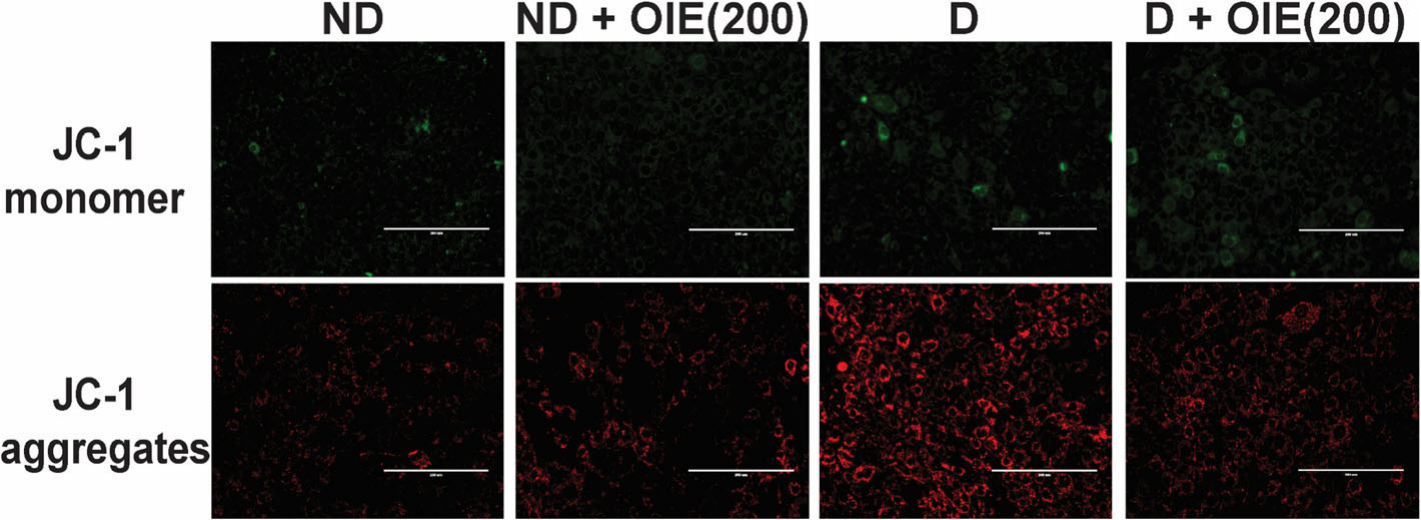
The effect of OIE on mitochondrial activity in 3 T3-L1 at 24 h. The cells were incubated with JC-1 fluorescence dye for 60 min. The images of cells labeled with JC-1 vitalized mitochondria were visualized using fluorescence microscopy, JC-1 monomer is visible as green and aggregates visible as red, scale bar, 200 μm. Two independent experiments (each run triplicate) were done. ND: non-differentiated cells (pre-adipocytes); ND + OIE(200): non-differentiated cells with OIE at 200 μg/mL (OIE-treated pre-adipocytes); D: differentiated cells with 0.1% DMSO (untreated-adipocytes), D + OIE(200): differentiated cells with OIE at 200 μg/mL (OIE-treated adipocytes)

**Fig. 8:**
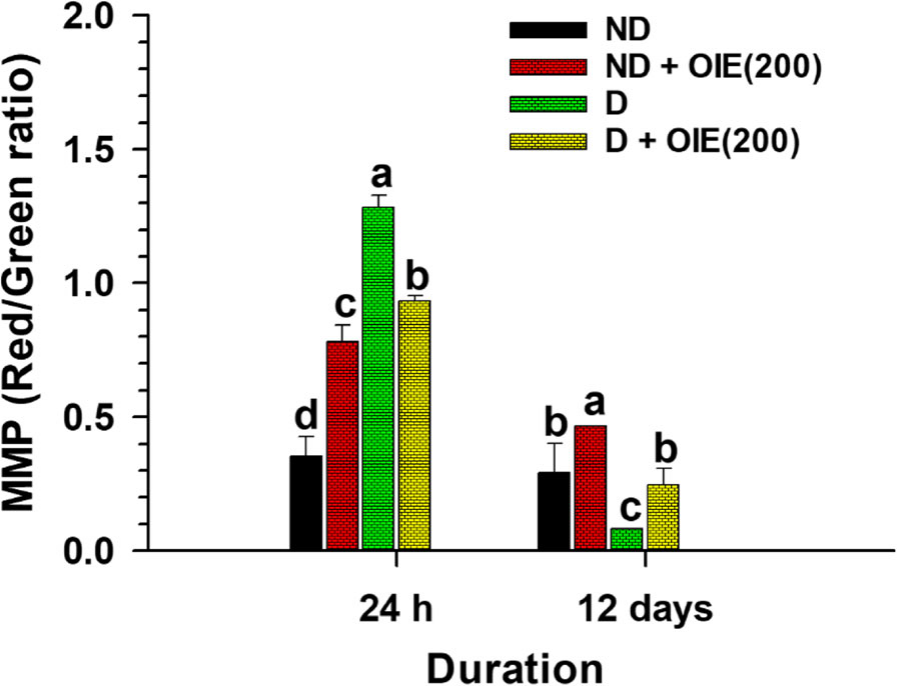
The effect of OIE on MMP level in 3 T3-L1 cells. The cells were incubated with JC-1 fluorescence dye for 60 min at different periods. Labeled cells were collected and analyzed using a flow cytometer with excitation 488 nm. Means ± SD value (*n* = 9) of three independent experiments is demonstrated. The data were reported as the ratio of red aggregates (FL-2 channel) to green monomer (FL-1 channel). ND: non-differentiated cells (pre-adipocytes); ND + OIE(200): non-differentiated cells with OIE at 200 μg/mL (OIE-treated pre-adipocytes); D: differentiated cells with 0.1% DMSO (untreated-adipocytes); D + OIE(200): differentiated cells with OIE at 200 μg/mL (OIE-treated adipocytes). Differences among groups were determined by one-way ANOVA followed by Tukey’s Post-hoc test, and the different superscript alphabets are significantly different from each other (*P* < 0.05)

**Fig. 9:**
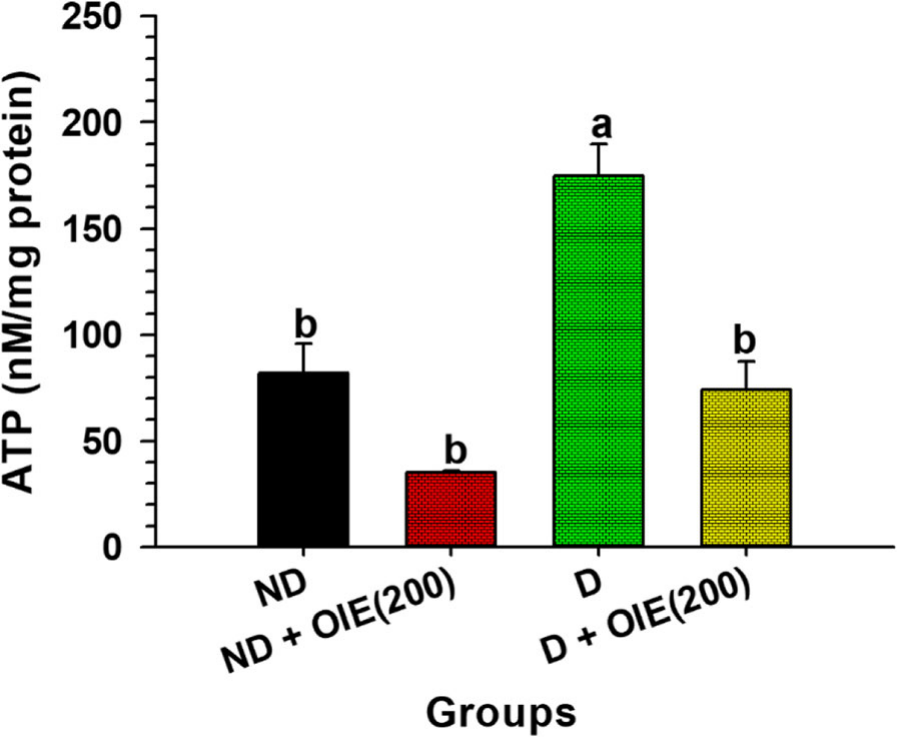
The effect of OIE on the ATP level at day 12. The level of ATP was determined by ATP bioluminescent. On day 12, the cells were incubated with an ATP releasing agent and transfer to the 96 well plates. The luciferase assay was added to the sample, then the amount of light emission was measured using a microplate luminometer. ATP concentration was determined from the standard curve. The protein concentrations in each group were used to normalize the ATP content (ηM/mg of protein). Means ± SD value (*n* = 9) of three independent experiments is indicated. ND: non-differentiated cells (pre-adipocytes); ND + OIE(200): non-differentiated cells with OIE at 200 μg/mL (OIE-treated pre-adipocytes); D: differentiated cells with 0.1% DMSO (untreated-adipocytes); D + OIE(200): differentiated cells with OIE at 200 μg/mL (OIE-treated adipocytes). Differences among groups were determined by one-way ANOVA followed by Tukey’s Post-hoc test; the different superscript alphabets are significantly different from each other (*P* < 0.05)

**Fig. 10:**
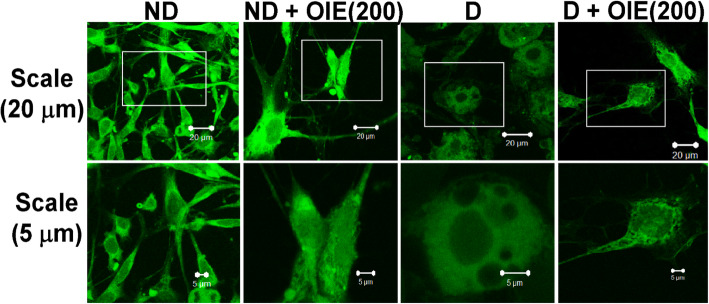
The effect of OIE on mitochondrial mass. On day 12, the cells were incubated with 200 nM of MitoTracker dyes for 45 min. Labeled cells were transferred to the Shandon cytofunnel chamber and mounted with the mounting media. Then, the cells were visualized by laser scanning microscopy (excitation 488 nm). Mitochondria labeled with MitoTracker dyes showed in green color, scale bar, 20 μm, and 5 μm. Two independent experiments (each run triplicate) were performed. ND: non-differentiated cells (pre-adipocytes); ND + OIE(200): non-differentiated cells with OIE at 200 μg/mL (OIE-treated pre-adipocytes); D: differentiated cells with 0.1% DMSO (untreated-adipocytes); D + OIE(200): differentiated cells with OIE at 200 μg/mL (OIE-treated adipocytes)

**Fig. 11:**
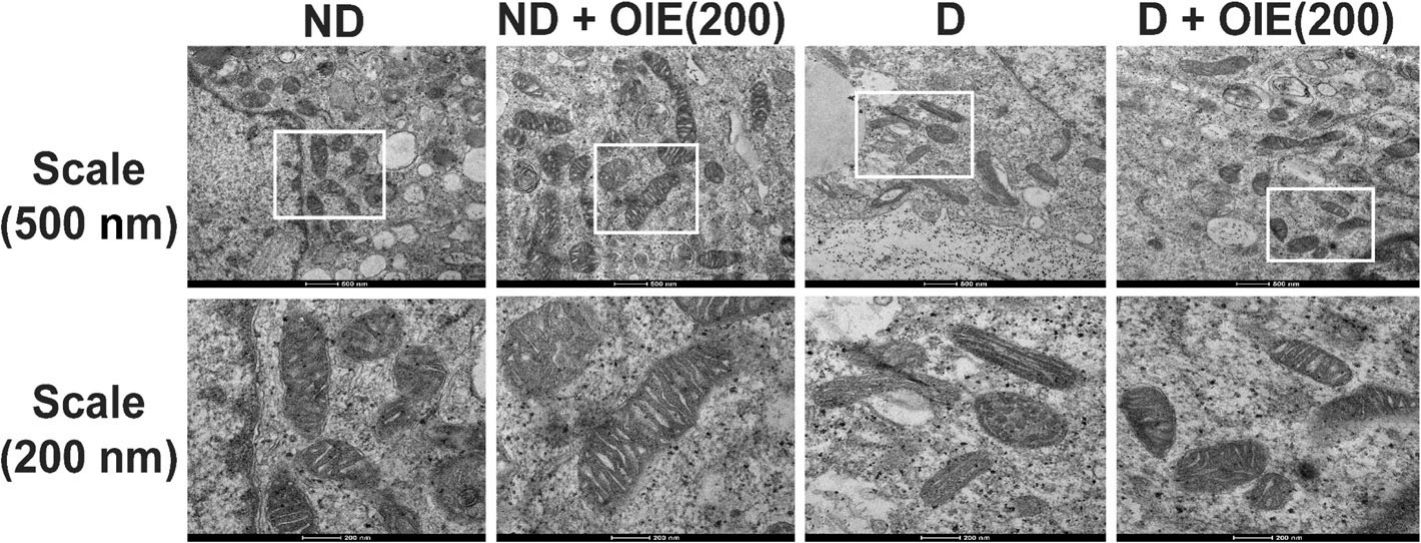
The effect of OIE on mitochondria morphology. On day 12, the cells were extracted, then fixed in 2.5% glutaraldehyde, and post-fixated with 1% Osmium tetroxide. The fixed sample was embedded in epoxy resin. The sample was sectioned at 60 nm with the ultra-microtome. The sections were stained with 2% uranyl acetate, followed by secondary staining with lead stain solution. The grids were observed under a TEM at 120 kV. The micrographs of mitochondria in 3 T3-L1 cells showed at scale bar 500 nm and 200 nm. Two independent experiments (each run triplicate) were operated. ND: non-differentiated cells (pre-adipocytes); ND + OIE(200): non-differentiated cells with OIE at 200 μg/mL (OIE-treated pre-adipocytes); D: differentiated cells with 0.1% DMSO (untreated-adipocytes); D + OIE (200): differentiated cells with OIE at 200 μg/mL (OIE-treated adipocytes)

The effect of the OIE on mitochondrial activity was evaluated by the MMP, intracellular ATP production, mitochondrial mass, and morphology determination. The JC-1 was used for monitoring the mitochondrial membrane. When the dyes enter into the cytoplasm of cells, it exhibits a green fluorescence color called the JC-1 monomer. However, when the dyes enter into the mitochondria, it will accumulate and start forming reversible complexes called JC-1 aggregates. These JC-1 aggregates exhibited excitation and emission in the red spectrum. Fluorescence staining showed that JC-1 monomer (green) and JC-1 aggregates (red) were displayed in all samples (Fig. 7). However, JC-1 aggregates in OIE-treated-ND, OIE-treated-D, and ND groups were less pronounced compared to the D (untreated-adipocytes) group (Fig. 7). This result is consistent with the flow cytometry data regarding MMP. The increase of MMP (red/green fluorescence ratio) was demonstrated in untreated adipocytes, while treatment with 200 μg/mL at 24 h significantly reduced the ratio (*P* < 0.05, Fig. 8). However, a noticeable reduction of MMP was found in all samples at day 12 compared to 24 h, especially in untreated adipocytes. Furthermore, the cellular ATP level was determined on day 12; the highest intracellular concentration of ATP level was found in untreated adipocytes. It was significantly reduced in adipocytes treated with OIE (*P* < 0.05, Fig. 9). MitoTracker dyes have been used for detecting a mitochondrial mass. This fluorescent dye is linking to thiol groups in the mitochondria, and it produces green fluorescence under excitation 488 nm. The results showed that untreated adipocytes displayed a decrease in the intensity of the labeling mitochondrial (Fig. 10), whereas the intensity seemed to increase with longer treatments with 200 μg/mL of the OIE. This observation was similar to pre-adipocytes alone or treated with OIE. This study explored the morphology of mitochondria by TEM [18]. The results indicated that mitochondria of pre-adipocytes with and without-OIE were gathered around the nucleus, and the morphology was mainly short, but the cristae were clearly observed (Fig. 11). In contrast, the mitochondria of untreated adipocytes had become a more condensed, slender shape with reduced cristae mitochondria and uniformly distributed in the cytoplasm. While OIE-treated adipocytes could recover mitochondria mass and morphology almost the same as untreated pre-adipocytes. These results suggest that mitochondria play an important role during 3 T3-L1 differentiation. During the adipocyte differentiation process of untreated pre-adipocytes at 24 h, the cells had an increase in MMP. Whereas OIE-treated adipocytes showed significantly lower and higher MMP levels compared to the OIE-untreated adipocytes at 24 h and day 12, respectively (*P* < 0.05, Fig. 8). However, on day 12, the ATP level of OIE-treated adipocytes was significantly lower compared to non-treated adipocytes (*P* < 0.05, Fig. 9).

## Discussion

Previous studies reported that OIE had an anti-adipogenic effect through inhibiting the expression of PPARγ along with a decrease in adiponectin and FAS production [8, 9]. In this study, we investigated the impact of the OIE on cell cycle, glucose metabolism, and mitochondria. OIE at doses of 50–200 μg/mL blocked the cell cycle on the G1/S stage at 24 h, and this was subsequently confirmed by a significant decrease in the expression of Cdk2, a protein involved in the transition of G1/S to S/G2. Phytochemicals, especially flavonoids, have been shown to processes adipogenesis suppressive in 3 T3-L1 cells. Pretreatment of 3 T3-L1 adipocytes with flavonoids such as baicalein and chrysin suppressed adipogenesis by inducing cell cycle arrest in the G0/G1 phase [19, 20]. In this study, LC-MS analysis showed that baicalein is one of the major components found in OIE. The result of the LC-MS chromatogram at peak 21 (m/z 254) lead us to believe that it could be chrysin, which is consistent with the standard chrysin peak [13]. Thus, it is possible that baicalein and chrysin in OIE responsible for anti-adipogenesis activity by blocking the cell cycle, which is an important adipogenic event at the early stage of cell differentiation. As well as other studies reported that other flavonoids or other chemical compounds inhibited adipocyte differentiation through a similar mechanism [21, 22].

The OIE may influence the metabolism of cells during the differentiation process. In order to understand the effect of the OIE glucose uptake and metabolism, this study evaluated the level of tyrosine phosphorylation associated with the IR, GLUT4 expression, and glucose uptake. The results indicated that the protein tyrosine kinase activity was apparent in the differentiated cells with or without OIE at 24 h. However, on day 12, the phosphorylation of proteins with molecular weights (MW) corresponding to 181 and 164 kDa was diminished by treatment with OIE. Whereas, a protein with MW at 91 kDa was not different compared to the untreated adipocytes. The proteins with molecular masses of 181, 164, and 91 kDa correspond to the insulin markers IRS-2, IRS-1, and IR β-subunit, respectively [15, 16, 23]. These results suggest that OIE may influence the activity of insulin and, thus, the uptake of glucose. The increasing phosphorylation of IRS-1 and IRS-2 may activate many different signaling pathways, including phosphatidylinositol-3-kinase (PI3 kinase) and AKT pathway [24]. The activation of PI3 and AKT pathway has been suggested to be a part of the signal transduction pathway for insulin-induced GLUT4 redistribution [18]. This study found that GLUT4 was slightly expressed in the same amount in all groups at 24 h. These observations are in substantial agreement with the study of Jackson and colleagues that at the early part of differentiation, the expression of GLUT4 is low but increases reach a peak at day 9 [25]. Similarly, in this investigation, GLUT4 was significantly increased and highly expressed to the cell membrane in untreated adipocytes at day 12. Whereas, the cells treated with OIE displayed a lower concentration of GLUT4. These findings are likely to believe that the cells are insensitive to insulin and may act on GLUT4 expression and subsequent glucose uptake. One suggestion is that OIE inhibits the phosphorylation of IRS-1 and IRS-2, leading to a decrease in GLUT4 protein expression compared to the untreated-adipocytes. If GLUT4 appearance is compromised, this would have an impact on glucose uptake and intracellular glucose concentrations. Moreover, in this study, we found that the OIE treated pre-adipocytes and adipocytes significantly increased glucose uptake level by approximately 2 folds compared to both untreated cells with no effect on GLUT4 expression. This may be explained as cells treated with OIE may use another facilitative transporter-like GLUT1 and is less dependent on GLUT4. In fact, the abundance of GLUT1 was found in 3 T3-L1 cells at the pre-adipocytes stage and diminished when cells become adipocytes [26]. Besides, in adipose tissue, GLUT1 is expressed along with GLUT4. However, GLUT4, which is insulin-dependent, are more dominantly expressed in adipose tissue than the GLUT1 [27]. Some of the antidiabetic drugs, such as rosiglitazone, enhanced glucose transport in the basal stage caused by an increase in the synthesis of GLUT1 RNA and protein [28]. Furthermore, thiazolidinediones in both basal and insulin stages increase the glucose uptake and cellular GLUT1 expression with no effect on GLUT4 expression [28, 29]. Another study also demonstrated that 3 T3-L1 cells treated with 50 μM of baicalein significantly suppressed the expression of the GLUT4 gene and decreased the glucose uptake level [30]. Moreover, it had been reported that 100 mg/kg body weight of chrysin inhibited insulin receptor 1 (IR1) gene expression and contributed to the reduction of IR1 protein in diabetic rats [31]. Also, Berberine is a major alkaloid component of *Rhizoma coptidis*, has shown a promising impact on the upregulation of GLUT-1 expression level by stimulating the extracellular signal-regulated kinases (ERK) pathway in 3 T3-L1 cells [32, 33].

Previous studies had demonstrated that during adipogenesis, mitochondria were present at number 19 folds greater than 3 T3-L1 pre-adipocytes [34]. Similarly, this investigation found that the level of MMP in untreated-adipocytes was significantly increased during the first 24 h compared to the pre-adipocytes (Fig. 8). However, on day 12, the MMP level of the untreated-adipocytes was dramatically decreased compared to the pre-adipocytes (Fig. 8), while the ATP level increased compared to pre-adipocytes (Fig. 9). Additionally, confocal microscopy revealed that the adipocytes’ mitochondria displayed pale green fluorescence, which represents the mitochondrial mass. Moreover, the morphology of adipocytes’ mitochondria was elongated. This finding is consistent with the previous study that during differentiation, the mitochondria continuously switch their morphology from globular to elongated mitochondria via mitochondrial fusion [12]. Accordingly, mitochondria undergo dynamic remodeling during differentiation, and thus, mitochondrial morphology and metabolism are changed. Although a massive increase in the number and activity of mitochondria beginning in the early adipogenesis [35]. This change is because mitochondria provide the essential substrate necessary for lipogenesis [36]. However, the study of Goldman and colleagues confirmed that mature adipocytes contained very few mitochondria compared to the cells undergoing in the differentiation process [37]. The decreasing of mitochondria content likely reflects the decreased need for lipogenesis in the mature white adipocyte. Likewise, the changing of mitochondria morphology could disrupt their function [34, 38, 39]. In the same way, the altered mitochondrial morphology in adipocytes was observed in this study. A previous study revealed that phytochemical compounds, including apigenin and quercetin, significantly protected mitochondrial alterations during ER stress in 3 T3-L1 adipocytes [40]. These findings provide evidence that apigenin and other flavonoids found in OIE may play a vital role in mitochondria protection. Thus, these findings lend support to the assumption that at the early phase of differentiation, untreated differentiated cells need a lot of ATP production for lipogenesis as shown by increasing of MMP level at 24 h, while the fusion of adipocytes mitochondria at day 12 takes effect to the mitochondria activity results in a decrease in the MMP level. Apart from this, an increasing ATP level in adipocytes might be explained hypothesizing that the mitochondria have been compromised, and then, the cell switches to glycolysis. However, ATP generated from the glycolysis pathway may not be enough for the cells. Thus, the cells try to keep homeostasis by increasing glucose uptake levels. On the contrary, OIE-treated adipocytes exhibited significantly lower and higher MMP levels at 24 h and 12 days, respectively, which use lower ATP like pre-adipocytes. Moreover, the mitochondria mass and morphology of the OIE-treated cells are virtually similar to pre-adipocytes mitochondria.

## Conclusion

In this study, we clarified that the anti-adipogenic activity of OIE was achieved through the inhibition of cell cycle progression and reduced the expression of IR protein resulted in a decrease in the glucose uptake level. Furthermore, OIE slowed down the mitochondrial activity of the early phase of cell differentiation, caused lipogenesis reduction, which was similar to the pre-adipocytes stage.

## Supplementary information

Additional file 1. The results of western blots analysis. **Figure S1.** Full range of the expression of cyclin-dependent kinase 2 (Cdk2) and beta actin (*β*-actin) in 3T3-L1 cells. **Figure S2.** Full range of the expression of glucose transporter4 (Glut4), protein tyrosine phosphorylation (PY20) and beta actin (*β*-actin) in 3T3-L1 cells. (ZIP 1234 kb)

## Data Availability

The datasets used and/or analyzed during the current study available from the corresponding author on reasonable request.

## Abbreviations

OIE: *Oroxylum indicum* fruit extract
*O. indicum*: *Oroxylum indicum*
Cdk2: Cyclin-dependent kinase 2
GLUT4: Glucose transporter4
PY20: Protein tyrosine phosphorylation
MMP: Mitochondrial membrane potential
IR: Insulin receptor
IRS: Insulin receptor substrate
ATP: Adenosine triphosphate
LC-MS: Liquid chromatography-mass spectrometry
DMSO: Dimethyl sulfoxide
ND: Non-differentiated cells (pre-adipocytes)
D: Differentiated cells with 0.1% DMSO (untreated-adipocytes)
D + OIE(200): Differentiated cells with OIE at 200 μg/mL (OIE-treated adipocytes)
2-NBDG: 2-deoxy-2-[(7-nitro-2,1,3-benzoxadiazol-4-yl)amino]-D-glucose
MCE: Mitotic clonal expansion
PI: Propidium iodide
ECL: Enhanced chemiluminescence
DMEM: Dulbecco’s Modified Eagle Medium
FBS: Fetal bovine serum
IBMX: 3-isobutyl-1-methylxanthine
DEX: Dexamethasone
PVDF: Polyvinylidene difluoride
HRP: Horseradish peroxidase
PBST: PBS with 0.1% Tween 20
BSA: Bovine serum albumin
FITC: Fluorescein isothiocyanate
DAPI: 4′,6-Diamidino-2-Phenylindole
PBS: Phosphate-buffered saline
JC-1: Fluorescent lipophilic carbocyanine dye
PI3 kinase: Phosphatidylinositol-3-kinase
ERK: Extracellular signal-regulated kinases
