# Supplementary figures and images for: An insight into anti-adipogenic properties of an Oroxylum indicum (L.) Kurz extract
Source: BMC Complement Med Ther. 2020 Oct 20;20:319. doi: 10.1186/s12906-020-03111-2 (PMC7576871; doi:10.1186/s12906-020-03111-2)

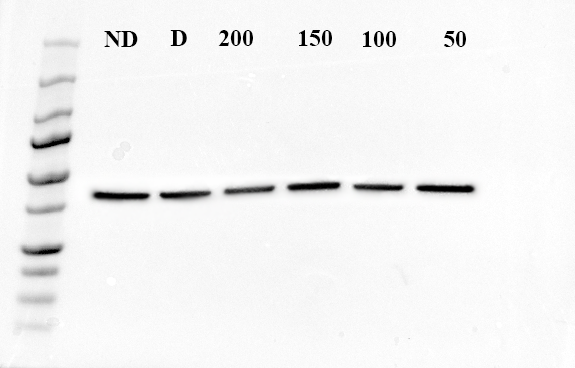

Supplement: Supplementary file 1 — The results of western blots analysis. Figure S1. Full range of the expression of cyclin-dependent kinase 2 (Cdk2) and beta actin (β-actin) in 3T3-L1 cells. Figure S2. Full range of the expression of glucose transporter4 (Glut4), protein tyrosine phosphorylation (PY20) and beta actin (β-actin) in 3T3-L1 cells. (ZIP 1234 kb) [file 12906_2020_3111_MOESM1_ESM.zip › Figure S1 (Beta-actin)R1.tif]

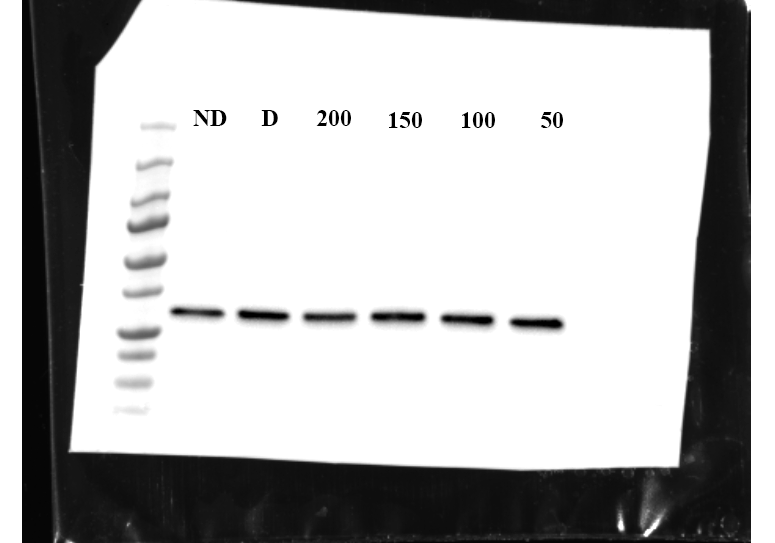

Supplement: Supplementary file 1 — The results of western blots analysis. Figure S1. Full range of the expression of cyclin-dependent kinase 2 (Cdk2) and beta actin (β-actin) in 3T3-L1 cells. Figure S2. Full range of the expression of glucose transporter4 (Glut4), protein tyrosine phosphorylation (PY20) and beta actin (β-actin) in 3T3-L1 cells. (ZIP 1234 kb) [file 12906_2020_3111_MOESM1_ESM.zip › Figure S1 (Cdk2)R1.tif]

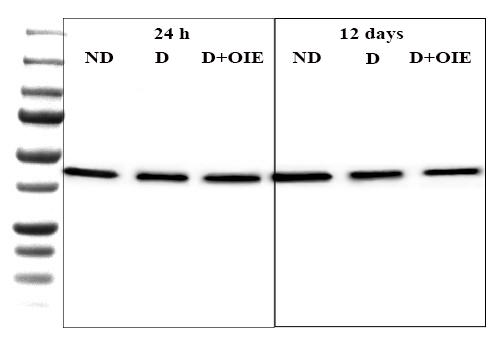

Supplement: Supplementary file 1 — The results of western blots analysis. Figure S1. Full range of the expression of cyclin-dependent kinase 2 (Cdk2) and beta actin (β-actin) in 3T3-L1 cells. Figure S2. Full range of the expression of glucose transporter4 (Glut4), protein tyrosine phosphorylation (PY20) and beta actin (β-actin) in 3T3-L1 cells. (ZIP 1234 kb) [file 12906_2020_3111_MOESM1_ESM.zip › Figure S2 (Beta-actin)R1.tif]

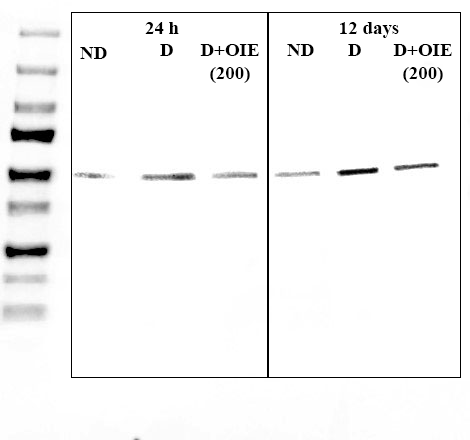

Supplement: Supplementary file 1 — The results of western blots analysis. Figure S1. Full range of the expression of cyclin-dependent kinase 2 (Cdk2) and beta actin (β-actin) in 3T3-L1 cells. Figure S2. Full range of the expression of glucose transporter4 (Glut4), protein tyrosine phosphorylation (PY20) and beta actin (β-actin) in 3T3-L1 cells. (ZIP 1234 kb) [file 12906_2020_3111_MOESM1_ESM.zip › Figure S2 (GLUT4)R1.tif]

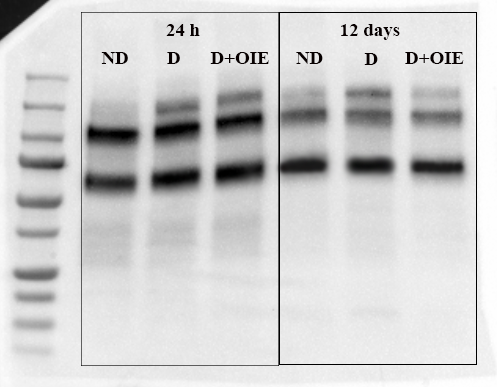

Supplement: Supplementary file 1 — The results of western blots analysis. Figure S1. Full range of the expression of cyclin-dependent kinase 2 (Cdk2) and beta actin (β-actin) in 3T3-L1 cells. Figure S2. Full range of the expression of glucose transporter4 (Glut4), protein tyrosine phosphorylation (PY20) and beta actin (β-actin) in 3T3-L1 cells. (ZIP 1234 kb) [file 12906_2020_3111_MOESM1_ESM.zip › Figure S2 (PY20)R1.tif]
